# A predictive model for L-T4 dose in postoperative DTC after RAI therapy and its clinical validation in two institutions

**DOI:** 10.3389/fendo.2024.1425101

**Published:** 2024-08-20

**Authors:** Jian-Jing Liu, Zi-Yang Wang, Yuan-Fang Yue, Guo-Tao Yin, Li-Na Tong, Jie Fu, Xiao-Ying Ma, Yan Li, Xue-Yao Liu, Li-Bo Zhang, Qian Su, Zhao Yang, Xiao-Feng Li, Wen-Gui Xu, Dong Dai

**Affiliations:** ^1^ Department of Molecular Imaging and Nuclear Medicine, Tianjin Medical University Cancer Institute and Hospital, Tianjin, China; ^2^ National Clinical Research Center for Cancer, Tianjin Key Laboratory of Cancer Prevention and Therapy, Tianjin’s Clinical Research Center for Cancer, Tianjin, China; ^3^ Department of Nuclear Medicine, Tianjin Cancer Hospital Airport Hospital, Tianjin, China; ^4^ Department of Radiology, Qilu Hospital of Shandong University, Jinan, Shandong, China

**Keywords:** differentiated thyroid cancer, radioactive iodine therapy, levothyroxine, thyroid stimulating hormone, machine learning

## Abstract

**Purpose:**

To develop a predictive model using machine learning for levothyroxine (L-T4) dose selection in patients with differentiated thyroid cancer (DTC) after resection and radioactive iodine (RAI) therapy and to prospectively validate the accuracy of the model in two institutions.

**Methods:**

A total of 266 DTC patients who received RAI therapy after thyroidectomy and achieved target thyroid stimulating hormone (TSH) level were included in this retrospective study. Sixteen clinical and biochemical characteristics that could potentially influence the L-T4 dose were collected; Significant features correlated with L-T4 dose were selected using machine learning random forest method, and a total of eight regression models were established to assess their performance in prediction of L-T4 dose after RAI therapy; The optimal model was validated through a two-center prospective study (n=263).

**Results:**

Six significant clinical and biochemical features were selected, including body surface area (BSA), weight, hemoglobin (HB), height, body mass index (BMI), and age. Cross-validation showed that the support vector regression (SVR) model was with the highest accuracy (53.4%) for prediction of L-T4 dose among the established eight models. In the two-center prospective validation study, a total of 263 patients were included. The TSH targeting rate based on constructed SVR model were dramatically higher than that based on empirical administration (Rate 1 (first rate): 52.09% (137/263) vs 10.53% (28/266); Rate 2 (cumulative rate): 85.55% (225/263) vs 53.38% (142/266)). Furthermore, the model significantly shortens the time (days) to achieve target TSH level (62.61 ± 58.78 vs 115.50 ± 71.40).

**Conclusions:**

The constructed SVR model can effectively predict the L-T4 dose for postoperative DTC after RAI therapy, thus shortening the time to achieve TSH target level and improving the quality of life for DTC patients.

## Introduction

The detection rate of thyroid nodules is high worldwide, with a prevalence of 20.43% in Chinese adults based on ultrasound examination findings of nodules larger than 0.5 cm in diameter, and approximately 8-16% of these nodules are malignant tumors (1). In recent years, the incidence of thyroid cancer significantly increased in China, with the majority being differentiated thyroid cancer (DTC), including papillary carcinoma and follicular carcinoma. Although DTC has a relatively low mortality, the mortality of patients with recurrence is on the rise, posing a threat to their quality of life. Due to the low mortality and mortality associated with DTC, standardized clinical management for DTC, including diagnosis, treatment, and prognostication is of necessity (2, 3).

Thyroid stimulating hormone (TSH) suppression therapy is an essential component of the treatment regimen for intermediate and high-risk DTC. It effectively reduces the risk of recurrence and mortality, thus improves the disease-free survival and quality of life for DTC (4–6). Both international and domestic guidelines recommend levothyroxine (L-T4) as the preferred oral medication for TSH suppression therapy, with a dosage of 1.5-2.5 µg/kg/day. The initial dosage may vary according to age and comorbidities (7, 8). It is known that both insufficiency and excessiveness in dosage of L-T4 can lead to adverse effects. Study from Paolo Miccoli et al. (9) also highlighted the importance of timely administration of optimal initial dose of L-T4 in DTC patients after total thyroidectomy. However, it is difficult for physician to prescribe the optimal dose of L-T4 at the very beginning because of the presence of various and unstable interfering factors affecting thyroid hormone levels. Moreover, the current guidelines lack explicit recommendations for the optimal initial dosage of L-T4, leading clinicians to rely on individual experience dosages. Thus, to standardize the clinical administration of L-T4 for DTC patients is in urgently needed (10). Though proposed mathematical models and solutions has been proposed by previous investigations to address this clinical issue (9, 11–16), significant discrepancies still exist, and a consensus has not yet been achieved.

**Figure 1:**
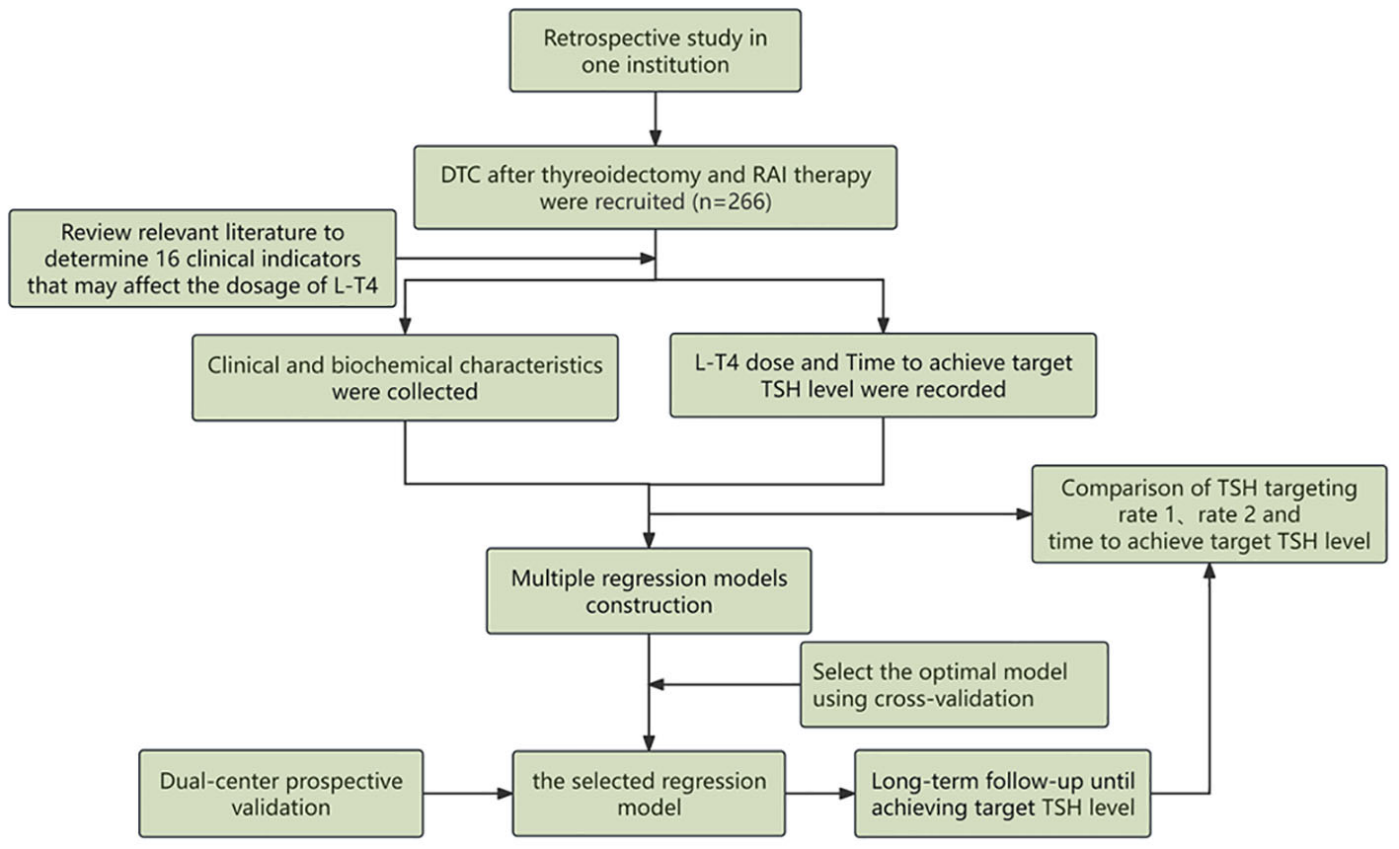
The general flowchart of this study, including construction of the predictive regression model in the retrospective cohort and validation of the model by two institutions in the prospective cohort.

In addition to TSH suppression therapy, radioactive iodine (RAI) treatment is also an important therapeutic approach for intermediate and high-risk DTC patients. RAI therapy is able to effectively eliminate residual thyroid tissue or metastatic lesions, which is beneficial to the clinical prospect of DTC patients after thyroidectomy. However, the demand for exogenous thyroid hormone (L-T4) is simultaneously increased for DTC patients with RAI therapy after thyroidectomy. Unfortunately, studies focused on this specific population of DTC were rare. In this study, we aimed to utilize machine learning methods to construct a predictive model for the optimal dosage of L-T4 in DTC patients after RAI treatment and prospectively validate its performance in two institutions. The flow chart of this study is drawn in **Figure 1**. The application of this developed model is expected to shorten the time required to achieve target TSH levels, thus alleviate the economic burden for DTC patients and finally improve their quality of life and clinical outcomes.

## Materials and methods

### Patient inclusion

This study consists of retrospective analysis and prospective analysis. In the retrospective analysis, data were collected from a total of 266 high/intermediate-risk DTC patients in our institution between November 2019 and November 2020 were collected, and all of them underwent TSH suppression therapy after thyreoidectomy and RAI therapy and achieved target TSH level. It is worth mentioning that, postoperative RAI therapy was administrated after a discontinuation of L-T4 for 3-4 weeks according to the guidelines from the American Thyroid Association (ATA), and no recombinant human TSH was used for all the included DTC patients. Of these patients, 78 were male and 188 were female, with a mean age of 40 ± 11.5 years. In the prospective analysis, a total of 263 DTC patients from two institutions were consecutively administered levothyroxine sodium tablets (brand name: L-T4, manufactured by Merck GmbH, Germany, approval number: H20140052, dosage: 50 µg) after RAI treatment. According to the 2015 guidelines from the ATA, the criteria for success of TSH suppression therapy after RAI treatment were as follows [7]: For postoperative DTC patients who were within 1 year of completion of surgery and/or RAI treatment, TSH suppression therapy was administrated to achieve target TSH levels which are TSH < 0.1 mU/L for high-risk group or TSH within 0.1-0.5 mU/L for intermediate-risk group, with both free triiodothyronine (FT3) and free thyroxine (FT4) levels within the normal range. Exclusion criteria in this study were as follows: [1] Patients with confirmed metastatic lesions based on imaging findings; [2] Patients younger than 18 years or older than 60 years; [3] Patients who became pregnant within one year after surgery; [4] Patients with severe cardiovascular or cerebrovascular diseases, or severe liver or kidney dysfunction. The study was reviewed and approved by the Institutional Ethics Committee, then being conducted in accordance with the Declaration of Helsinki. The written informed consent was obtained for the included DTC patients in the prospective cohorts, but the consent was waived for not for the patients in the retrospective cohorts.

### Clinical information collection

As shown in **Table 1**, a total of 16 clinical and biochemical characteristics that may influence L-T4 dosage in DTC patients were identified for clinical information collection. All included DTC patients were treated with L-T4 for TSH suppression therapy. In the retrospective analysis, the dosage of L-T4 after RAI therapy was prescribed empirically based on BMI, with 100 µg/d for patients within the normal BMI range and 1.5-2.2 µg/kg/d for patients outside the normal range. The dosage was empirically reduced for elderly patients, those with coronary artery disease and those with severe or long-standing hypothyroidism. After regular intake for 4-6 weeks, were conducted, and the dosage of L-T4 was adjusted by the physician based on thyroid function tests until achieving target TSH level.

**Table 1:** The 16 clinical and biochemical characteristics of 266 DTC finally included in the retrospective study.

	N (%)/M+SD
Sex	
Male	78 (29.3%)
Female	188 (70.7%)
Pausimenia	
Yes	46 (23.9%)
No	142 (76.06%)
Hashimoto’s thyroiditis	
Yes	71 (26.7%)
No	195 (73.3%)
Cardiovascular and cerebrovascular diseases	
Yes	27 (10.2%)
No	239 (89.8%)
Digestive system disease	
Yes	19 (7.1%)
No	247 (92.9%)
Combined with other malignancies	
Yes	4 (1.5%)
No	262 (98.5%)
Family history of malignancy	
Yes	22 (8.3%)
No	244 (91.7%)
Age (year)	41.24 ± 11.59
Weight (kg)	68.44 ± 12.92
Height (cm)	166.47 ± 7.85
Body mass index, BMI	24.57 ± 3.45
Body Surface Area, BSA (m2)	2.36 ± 0.19
Hemoglobin, HB (g/L)	139.51 ± 20.51
Mean corpuscular volume, MCV (fl)	86.92 ± 13.20
Systolic pressure/Diastolic pressure (mmHg)	125.60 ± 18.86/82.68 ± 12.29
Post-operative parathyroid hormone (pmol/L)	3.67 ± 2.43

### Clinical feature selection and mathematical model construction

First, the significance of each baseline clinical features was determined using a random forest model. Second, a new random forest model was trained using the entire retrospective dataset, including a total of 16 baseline clinical features, and the out-of-bag mean square error was recorded. Then, the least significant baseline clinical feature was removed and the model was retrained with the out-of-bag mean square error recorded. Finally, the combination of baseline clinical features with the lowest out-of-bag mean square error was selected. In this investigation, eight regression models were built with the selected features as independent variables and the optimal L-T4 dose as the dependent variable, including Linear Regression (LiR), Poisson Regression (PR), Random Forest Regression (RFR), Support Vector Regression (SVR), Ridge Regression (RR), Lasso Regression (LaR), K Neighbors Regression (KNR), and Gamma Regression (GR). Among the constructed eight regression models, the model with the highest accuracy was selected as the optimal model using 5-fold cross-validation. When the absolute difference between the predicted optimal L-T4 dose by the model and the practical L-T4 dose for achieving target TSH level after RAI therapy was less than 12.5ug, the prediction was considered correct.

### Dual-center prospective validation

From July 1, 2021 to February 28, 2022, a dual-center prospective validation was conducted in our institution (Center 1, n=110) and Tianjin Cancer Hospital airport hospital (Center 2, n=214). The inclusion criteria and exclusion criteria were listed in the following. Inclusion: [1] Patients who were pathologically confirmed as DTC and underwent total thyroidectomy; [2] DTC patients classified as intermediate or high risk of recurrence based on the 2015 ATA guidelines; [3] DTC patients receiving RAI therapy for remnant ablation after thyroidectomy; [4] The ages (year) of DTC patients were within a range from 18 to 60; [5] DTC patients who complied with the proposed L-T4 treatment and regular thyroid function tests; [6] DTC patients who achieved the target TSH level after several rounds regulation of L-T4 treatment. Exclusion criteria: [1] DTC patients who underwent subtotal thyroidectomy or had significant residual thyroid tissue according to thyroid imaging; [2] DTC patients with confirmed lymph node and/or lung and/or bone metastases based on imaging findings; [3] DTC patients with severe hepatic or renal impairment who cannot tolerate RAI therapy; [4] Patients who became pregnant during the follow-up after RAI therapy; [5] DTC patients with concomitant coronary heart disease or severe or long-term hypothyroidism; [6] DTC patients without with follow-up information due to poor compliance.

**Figure 2:**
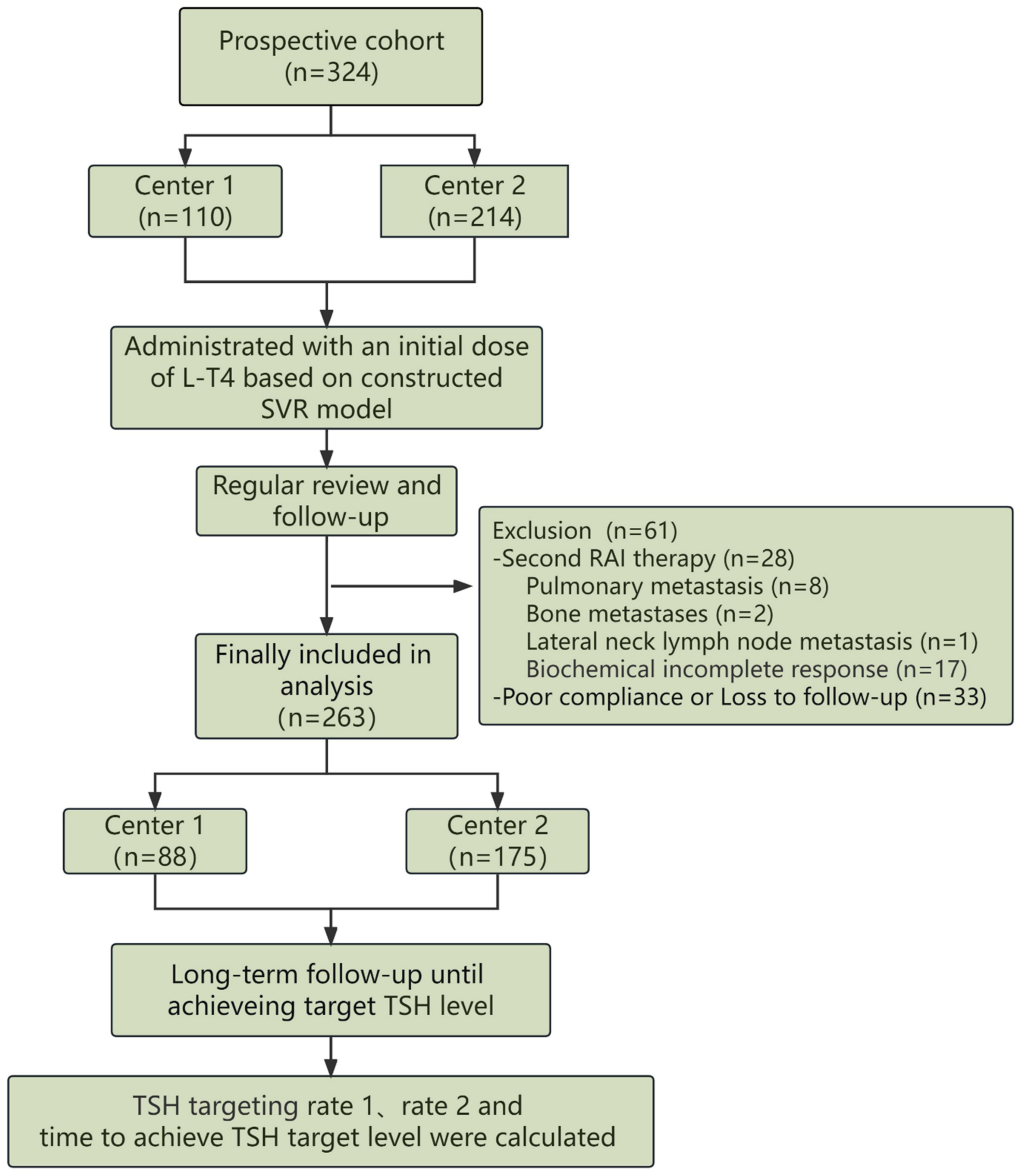
The detailed scheme regarding validation of the developed model in a two-center prospective study was depicted, including inclusion, L-T4 administration and evaluation of the predictive model.

All consecutively enrolled patients in the prospective study were administrated with a dose of L-T4 at the second day of RAI therapy based on the prediction by the selected regression model. The time needed to achieve target TSH level was recorded, including the first follow-up TSH targeting rate (referred to as rate 1) and the cumulative TSH targeting rate at the second follow-up (referred to as rate 2). Additionally, the mean time to achieve target TSH level was calculated. The scheme of the dual-center prospective validation is depicted in **Figure 2**.

### Statistical analyses

Statistical analysis was performed using SPSS 22.0 software, and differences between groups with a significance level of P < 0.05 was considered statistically significant. Quantitative variables with a normal distribution were presented as mean ± standard deviation (x ± S), and comparisons were conducted using T-test. Categorical variables were presented as numbers and percentages, and comparisons between groups were performed using the chi-square test. All feature selection and regression model construction were implemented using scikit-learn software version 0.23.2 based on Python 3.7.6 (https://scikit-learn.org/stable).

## Results

### Characterization of a total of 16 clinical and biochemical factors for DTC patients retrospectively enrolled in the study

A total of 266 DTC patients in our institution were ultimately included in the retrospective analysis for model construction, and 16 clinical and biochemical factors are characterized in **Table 1**. By Empirical administration, only 28 DTC patients achieved target TSH level one month after L-T4 therapy. In other words, the TSH targeting rate is 10.53% (28/266) in the first round of follow up. The cumulative TSH targeting rate was 53.38% (142/266) in the second round of follow-up two months after L-T4 therapy. In the long-term follow-up, it was revealed that the average time for all DTC patients to achieve target TSH level after L-T4 therapy was 115.50 ± 71.40 days. Subgroup analysis according to gender showed that the rate 1 was 6.41% (5/78) and the rate 2 was 48.72% (38/78) for the male subgroup, with an average time of 127.8 ± 80.10 days to achieve target TSH level; In contrast, the rate 1 was 12.23% (23/188) and the rate 2 was 55.32% (104/188) for the female subgroup, with an average time of 110.40 ± 67.20 days to achieve target TSH level. However, the differences between the male subgroup and the female subgroup were not statistically significant (P > 0.05).

### Feature selection by machine learning and regressive model construction

**Figure 3:**
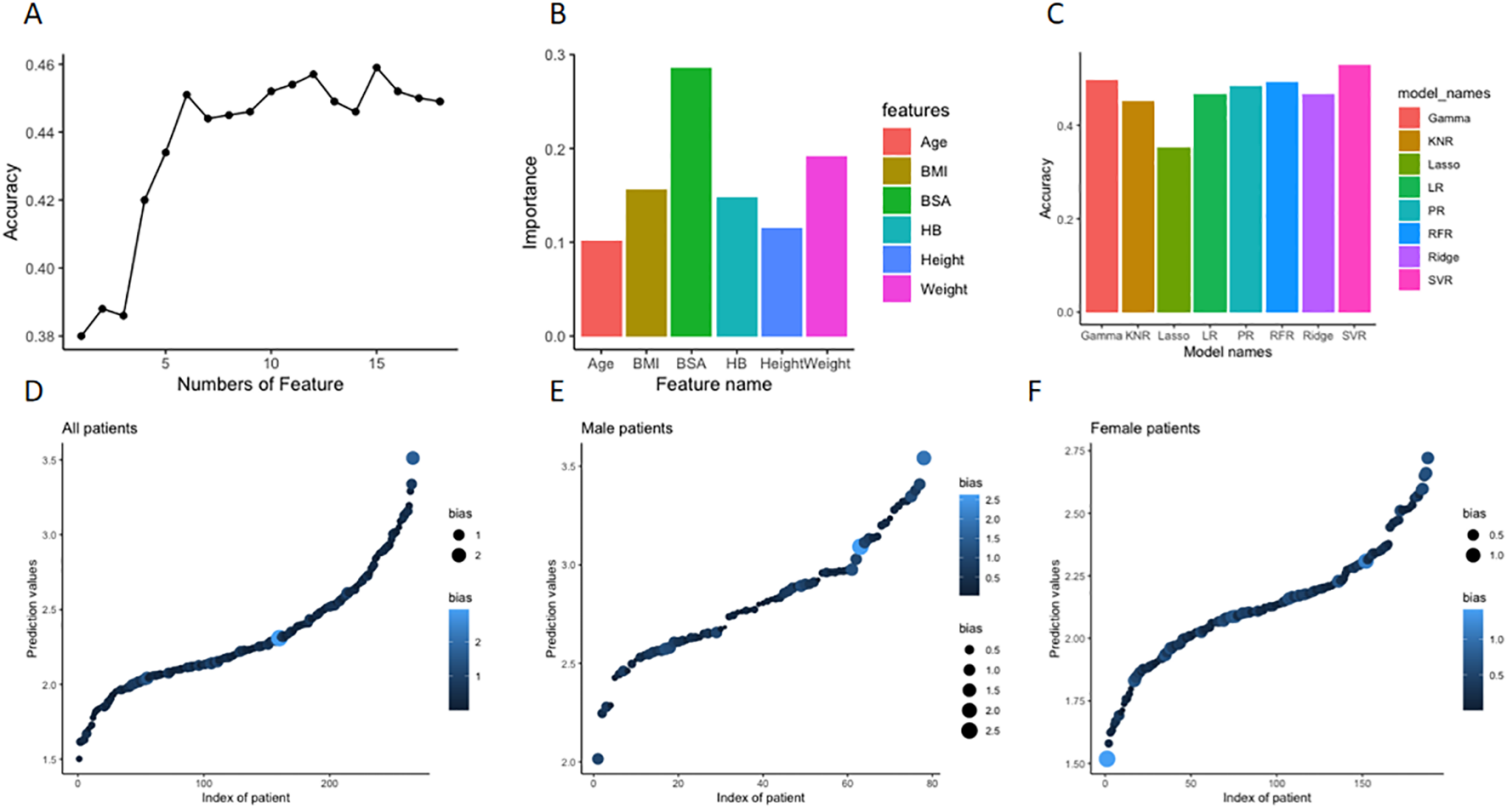
Feature selection by machine learning and regressive model construction for prediction of optimal L-T4 dose for DTC patients after resection and RAT therapy. **(A)** First, the significance of each baseline features (n=16) was determined using a random forest model. Then, the least significant feature was removed one by one until a combination of features with the lowest out-of-bag mean square error was selected. **(B)** The top 6 significant features, including body surface area (BSA), weight, hemoglobin, height, BMI, and age were finally selected, and the value of importance of each feature was depicted in the histogram. **(C)** Based on the selected 6 features, commonly used regression models were established to predict the optimal doses of L-T4. As demonstrated in the histograms, the predictive accuracies of the models were ranked from low to high. Among them, the highest SVR model achieved an accuracy of 53.4%. **(D)** The distribution of predicted doses of L-T4 by the constructed SVR model compared to the actual doses in empirical administration of L-T4 in the whole retrospective cohort. X axis is index of patient number, Y axis is the predicted dose (tablet) by the model. The size and color of dots represent the absolute deviation between the actual dose and the predicted dose. The bigger size and lighter color mean bigger deviation. **(E)** In the subgroup analysis according to gender, the distribution of predicted doses of L-T4 by the constructed SVR model compared to the actual doses in empirical administration of L-T4 in the male subgroup. **(F)** In the subgroup analysis according to gender, the distribution of predicted doses of L-T4 by the constructed SVR model compared to the actual doses in empirical administration of L-T4 in the female subgroup.

Machine learning random forest was used to determine the significance of each baseline clinical and biochemical features and then select the most optimal combination of multiple features with a high level of prediction accuracy. As demonstrated in **Figures 3A, B**, the top 6 significant features, including body surface area (BSA), weight, hemoglobin, height, BMI, and age, were finally selected. Using the selected clinical features as independent variables and the optimal doses of L-T4 when achieving target TSH level as the dependent variable, 8 commonly used regression models were established. Due to the limited sample size, 5-fold cross-validation was performed. As demonstrated, the predictive accuracies, ranked from low to high, were as follows: LaR, KNR, RR, RFR, GR, PR, LiR, and SVR model. Among them, the SVR model achieved an accuracy of 53.4% (**Figure 3C**). The distribution of predicted doses by the SVR model compared to the actual doses in empirical administration for the total included DTC patients is shown in **Figure 3D**. Subgroup analysis using the gender feature revealed that the accuracy of the SVR model was 35.9% for male subgroup and 60.64% for female subgroup (p < 0.01). The distributions of predicted doses by the SVR model compared to the actual doses in empirical administration for male subgroup and female subgroup are shown in **Figures 3E, F**, respectively.

### Dual-center prospective validation

The constructed SVR regression model was prospectively validated in two institutions. Originally, a total of 324 patients with DTC participated in this prospective study. During the follow-up, 28 patients who were recommended for repeat RAI therapy due to their poor biochemical incomplete response (BIR), poor structural incomplete response (SIR) or other clinical evaluations, were finally excluded in the prospective study. Specifically, the 28 DTC patients were composed of 8 patients with pulmonary metastasis, 2 patients with bone metastasis, 1 patient with lateral neck lymph node metastasis and 17 patients with poor biochemical response. Another 33 DTC patients who failed to comply to the proposed L-T4 treatment and follow-up were also excluded. Ultimately, a total of 263 DTC patients were finally included in the prospective analysis, with 88 cases from Center 1 and 175 cases from Center 2. The selected 6 clinical and biochemical features of DTC patients from both institutions and the predicted optimal L-T4 doses are presented in **Table 2**, indicating no significant differences between the two centers.

**Table 2:** Comparisons of characteristics and outcomes after L-T4 administration based on constructed SVR model between the two centers in the prospective study.

	Center 1	Center 2	T/χ^2^	P
N	88	175		
Sex			0.048	0.826
Male	25	52		
Female	63	123		
Age (year)	40.22 ± 10.49	40.49 ± 9.70	-0.202	0.840
Height (cm)	165.81 ± 7.94	165.03 ± 7.81	0.751	0.454
Weight (Kg)	72.23 ± 16.42	70.32 ± 16.39	1.358	0.176
Hemoglobin, HB (g/L)	139.74 ± 20.65	139.35 ± 19.63	0.145	0.885
Body mass index, BMI	26.52 ± 4.97	15.63 ± 4.47	1.424	0.158
Body Surface Area, BSA(m^2^)	1.91 ± 0.23	1.87 ± 0.24	1.332	0.184
Predicted optimal L-T4 doses (ug/d)	121.50 ± 20.00	117.00 ± 21.00	1.642	0.102
TSH targeting rate 1 (Rate 1)	51.14% (45/88)	52.57% (92/175)	0.048	0.826
TSH targeting rate 2 (Rate 2)	88.64% (78/88)	82.86% (147/175)	1.018	0.313
Time to achieve target TSH level (d)	59.13 ± 53.93	64.36 ± 61.30	-0.680	0.497

TSH, thyroid stimulating hormone.

**Figure 4:**
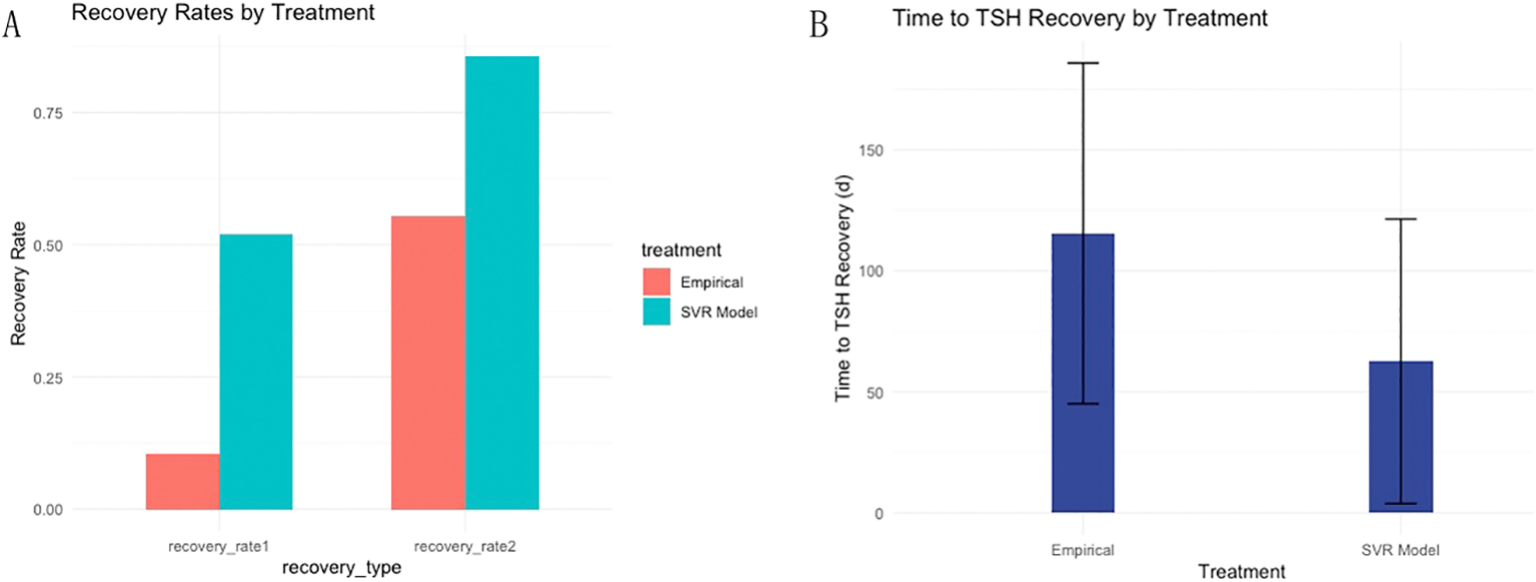
Dual-center prospective validation of the established SVR model in prediction of optimal L-T4 dose for DTC patients after resection and RAT therapy. **(A)** Compared to empirical dosing, L-T4 administration based on the predicted optimal dose by the constructed SVR model significantly enhanced both TSH targeting rate 1 and rate 2. **(B)** Furthermore, the average time to achieve target TSH level was dramatically reduced based on the dose prediction of L-T4 by SVR model in contrast with empirical dosing of L-T4.

Among the 263 patients finally assessed in the two-center prospective analysis, 137 patients achieved target TSH level one month after L-T4 treatment, resulting in a rate 1 of 51.14% (45/88) and 52.57% (92/175) in Center 1 and Center 2, respectively. For the remaining 126 patients who failed to achieve target TSH level at the first round of follow up, dose titration for L-T4 were executed during the second round of follow up, and another 88 DTC patients also achieved target TSH level. Thus, the rate 2 was 88.64% (78/88) in Center 1 and 82.86% (147/175) in Center 2. And. the average time to achieve target TSH level was 59.13 ± 53.93 days in Center 1 and 64.36 ± 61.30 days in Center 2. Compared to empirical dosing, L-T4 administration based on the predicted optimal dose by the constructed SVR model significantly enhanced both rate 1 (52.09% (137/263) vs 10.53% (28/266)) and rate 2 (85.55% (225/263) vs 55.38% (142/266)) (**Figure 4A**), and dramatically reduced the average time (days) (62.61 ± 58.78 vs 115.50 ± 71.40) to achieve target TSH level (**Figure 4B**). The subgroup analysis (**Table 3**) according to gender showed that in the whole prospective cohort, the rate 1 for male subgroup was 45.45% (35/77), with a rate 2 of 88.31% (68/77). For female subgroup, the rate 1 was 54.84% (102/186), with a rate 2 of 84.41% (157/186). Though the differences in TSH targeting rates between the female and male subgroups in the whole prospective cohort were not statistically significant, both the TSH targeting rate 1 and rate 2 in the male subgroup was significantly lower than that of the female subgroup for center 1. Moreover, the time taken to achieve target TSH level in the male subgroup was longer than that in female subgroup. Whereas, subgroup analysis according to gender revealed no significant differences for center 2.

**Table 3:** The subgroup analysis of 263 DTC according to gender in the prospective cohort.

		Male	Female	T/χ^2^	P
Center 1 (n=88)	N (%)	25 (28.41%/)	63 (71.59%)		
	Rate 1	20.00% (5/25)	63.49% (40/63)	13.549	< 0.0001
	Rate 2	84.00% (21/25)	90.48% (57/63)	0.745	0.388
	Time to achieve target TSH level (d)	85.40 ± 79.75	48.70 ± 35.07	3.010	0.003
Center 2 (n=175)	N (%)	52 (29.71%)	123 (70.29%)		
	Rate 1	57.69% (30/52)	50.41% (62/123)	0.778	0.378
	Rate 2	90.38% (47/52)	81.30% (100/123)	2.224	0.134
	Time to achieve target TSH level (d)	54.33 ± 48.21	68.60 ± 65.78	-1.412	0.160
The whole prospective cohort (n=263)	N (%)	77 (29.28%)	186(70.72%)		
	Rate 1	45.45%(35/77)	54.84% (102/186)	1.922	0.166
	Rate 2	88.31% (68/77)	84.41% (157/186)	0.671	0.413
	Time to achieve target TSH level (d)	64.42 ± 61.50	61.86 ± 57.92	0.320	0.749

TSH, thyroid stimulating hormone.

## Discussion

In the era of big data, an increasing number of machine learning algorithms have been applied in the healthcare environment in recent years. Our study recruited DTC patients who underwent RAI therapy and screened a total of 16 clinical and biochemical indicators that may affect the L-T4 dose to construct predictive models for optimal L-T4 administration. First, a retrospective analysis was conducted based on 266 DTC patients with TSH suppression therapy in our hospital. Second, a total of 6 indicators were finally selected to construct predictive models based on calculations of significance of factors by machine learning method named random forest, including BSA, weight, HB, height, BMI, and age. Then, eight regression prediction models were constructed to evaluate their predictive performance in optimal L-T4 dose. In the end, the SVR model with the highest predictive accuracy (53.4%) was selected in the following prospective analysis after cross-validation.

Noticeably, the constructed model was used for quantitative prediction of non-continuous variables rather than qualitative prediction of categorical variables, an accuracy of over 50% for model was considered markedly improved in comparison with that of empirical administration. Additionally, to illustrate the predictive performance of the constructed SVR model, the absolute deviation between the predicted and actual doses of L-T4 were shown in **Supplementary Figures S1, S2**. As indicated, when the dose deviation was limited to ≤ 25μg (L-T4 is a tablet, and clinically adjustable minimum dosage is 1/4 tablet, i.e., 12.5ug), the percentage of DTC patients with a predicted dose of ≤ 25μg for L-T4 is up to 80.45% (214/266), which is considered as a clinically acceptable performance for a potential application in clinical practice. In addition to retrospective analysis, the model was also prospectively validated in two centers. The median time taken to achieve target TSH level was 30 days based on predicted L-T4 dose by constructed SVR model, which was significantly shorter than that of empirically administration of L-T4 in the retrospective study. The TSH targeting rate 1 was 52.09%, and TSH targeting rate 2 was 85.55%, both of which were significantly higher than those in the empirical administration group from the retrospective cohort.

Brun et al. (16) pioneered the development of a computer program as a decision aid tool (DAT) to simulate L-T4 dosage and customized it for individual patients. However, this study only included retrospective data from 46 post-thyroidectomy patients. Zaborek et al. (12) selected 7 factors (age, sex, weight, BMI, preoperative TSH level, iron and multivitamin supplementation) to construct multiple regression models based on machine learning to predict the optimal initial dose of L-T4 for total thyroidectomy patients. The Poisson regression model developed in the study had the highest accuracy (64.8%), but this study was only limited to patients with benign thyroid diseases. Although a domestic study (11) with respect to the initial L-T4 dose after surgery in DTC patients was also conducted, the number of cases included in the study and the relevant indicators selected (age and BMI) were both limited, and only a single logistic regression model was constructed. Previous studies on L-T4 dosage only focused on relatively few influencing factors. Olobuwale et al. (17) and Jin et al. (15) developed two different weight-based schemes to calculate the optimal L-T4 dosage for thyroidectomy patients. The unsatisfactory results indicated that weight alone is not the sole factor in determining LT-4 dosage (18), Mistry et al. (19), Ojomo et al. (20), Di Donna et al. (21) and Elfenbein et al. (14) proposed incorporating age, gender, weight and BMI into the calculation for L-T4 dosage. As reported in their investigations, over 60% of DTC patients achieved normal thyroid function status at their first follow-up, indicating a significant improvement in the calculation effectiveness. Al Dhahiri et al. (13) identified BSA as an independent predictor of L-T4 dosage and developed two complex formulas (one polynomial and one linear) to predict the dosage of L-T4 for post-thyroidectomy patients, with accuracy rates of 65.8% and 51.3%, respectively. However, due to the complexity of the formulas, their clinical applicability remained limited.

The performance of SVR model in our study was acceptable compared to that of the Poisson regression model (64.8%) established by Zaborek et al. (12). Because the patients included in Zaborek’s study were limited to those with benign thyroid diseases who only required thyroid hormone replacement therapy with a wide range of TSH control (0.45-4.50 mIU/mL). The residual hypothalamic-pituitary-thyroid system was still able to regulate the thyroid hormone levels in the body, to some extent compensating for the dosage calculation errors of predictive models. Whereas, the cases in our study were intermediate to high-risk DTC patients who underwent postoperative RAI therapy, which led to effective elimination of almost all thyroid tissue and functional lesions in the body. Thereby, a higher demand for accurately determined L-T4 doses with less room for error in the predictive model was required for the TSH suppression therapy. Subgroup analyses according to gender were also performed. The accuracy of SVR model for the female subgroup was significantly higher than that for in the male subgroup (60.64% vs 35.9%, p < 0.01). The relative larger sample size of the female subgroup compared to that of male subgroup may contribute to the bias in the prediction efficacy, or the SVR was actually more suitable for the female group, which needed to be further confirmed.

Additionally, though previous study have reported L-T4 dose selection based on machine learning model for the treatment of postoperative hypothyroidism with a larger sample size (22), our study have some innovations deserved to be addressed. First, our study focused on DTC patients after resection and RAI, while other studies were mainly restricted to postoperative DTC; Second, the machine learning model in our study was constructed based on a total of 6 baseline clinical characteristics, whereas AI models from others only included limited clinical features; Then, a total of eight regression models were developed in the study to choose the optimal model among them with the highest prediction accuracy; Last but not least, Our study consist of retrospective cohort and prospective cohort, which allow us to test the constructed model in two-center prospective study.

Though innovations mentioned above, some limitations associated with this study existed. First, the sample size in the retrospective study used for model training was relatively small, and a larger number of cases were needed to further improve the predictive model. Additionally, the population included in this study is only limited to intermediate and high-risk DTC patients with RAI therapy. It is necessary to further optimize the model by applying this method to perform analysis with a larger sample size for low-risk DTC patients or patients with benign thyroid disease. Meanwhile, to further shorten the time to achieve TSH target level in clinical practice, a more standard supervision protocol for dose adjustment is urgently needed. Second, although this study screened a total of 16 clinical and biochemical factors that could affect the L-T4 dose and selected six indicators for model construction, the factor of gender was not chosen for model building. However, subgroup analysis according to gender showed significant differences in the predictive performances of SVR models for optimal L-T4 dose. Further multicenter prospective studies are needed to improve the analysis of various subgroups.

## Conclusion

The constructed SVR model can effectively predict the L-T4 dose for postoperative DTC after RAI therapy, thus shortening the time to achieve target TSH level and improving the quality of life for DTC patients.

## Data Availability

The original contributions presented in the study are included in the article/**Supplementary Material**. Further inquiries can be directed to the corresponding authors.
