# Supplementary material for: A predictive model for L-T4 dose in postoperative DTC after RAI therapy and its clinical validation in two institutions
Source: Front Endocrinol (Lausanne). 2024 Aug 20;15:1425101. doi: 10.3389/fendo.2024.1425101 (PMC11368713; doi:10.3389/fendo.2024.1425101)

**Supplementary materials**

**Figure S1**

The distribution of predicted doses of L-T4 by the constructed SVR model compared to the actual doses of L-T4 in empirical administration in the whole retrospective cohort. X axis is index of patient number, Y axis is a ratio (100%) of (actual dose - predicted dose) to actual dose. The size and color of dots represent the absolute deviation between the actual dose and the predicted dose. The bigger size and lighter color mean bigger deviation.


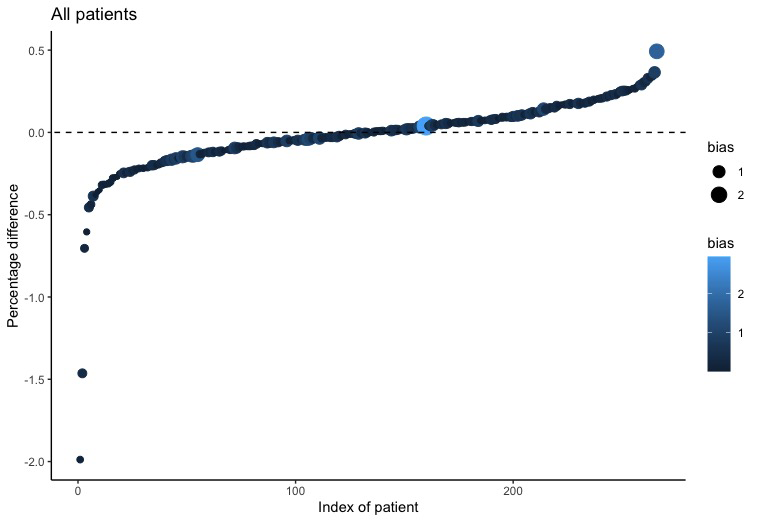


**Figure S2**

A bar plot for the number distribution of DTC patients with different ranges of predicted L-T4 dose. As indicated, when the dose deviation was limited to ≤ 25μg (L-T4 is a tablet, and clinically adjustable minimum dosage is 1/4 tablet, i.e., 12.5ug), the percentage of DTC patients with a predicted dose of ≤ 25μg for L-T4 is up to 80.45% ((141+73)/266).


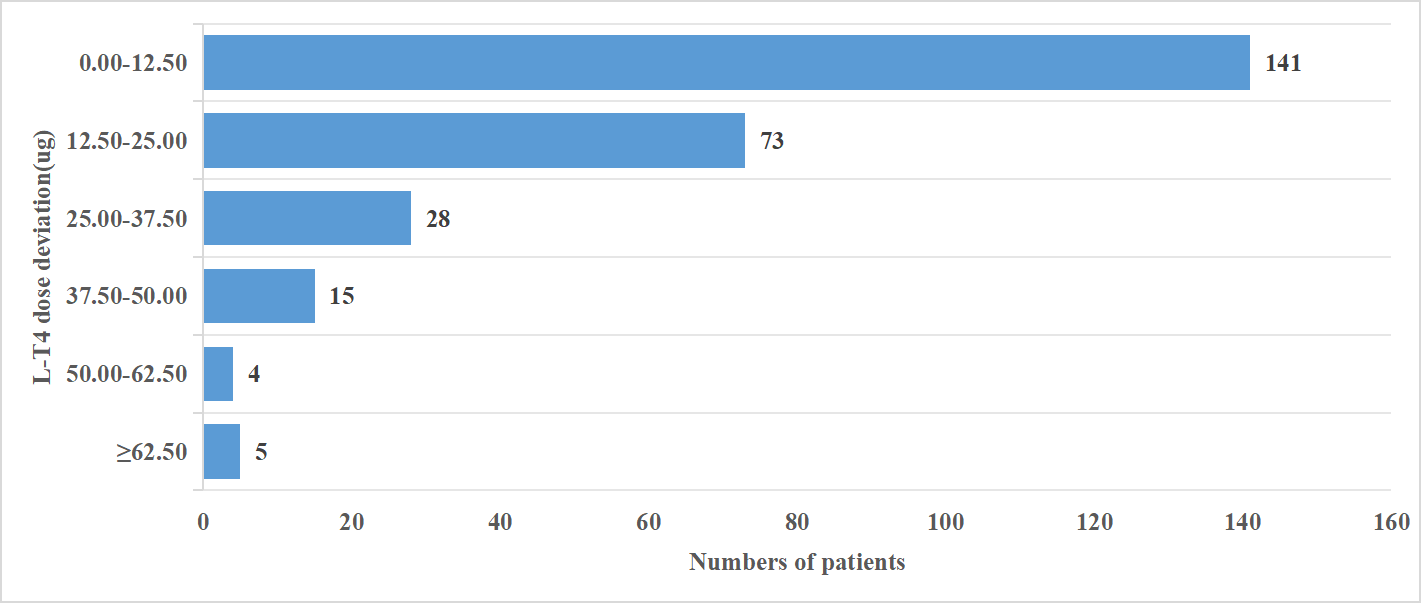

Supplement: Supplementary file 1 [file DataSheet1.doc]
